# Volvulus of the Transverse Colon: A Report of Two Cases and Review of Literature

**DOI:** 10.7759/cureus.86301

**Published:** 2025-06-18

**Authors:** Mohamed Ahmed, Ahmed Allawi, Sarmad Mohammed Salih, Garnet Vanterpool, Danya Auda

**Affiliations:** 1 Surgery, AdventHealth, Tampa, USA; 2 Surgery, University of California, Riverside, USA; 3 Colorectal Surgery, AdventHealth, Orlando, USA; 4 General Surgery, Östersund General Hospital, Östersund, SWE; 5 Surgery, Oxford University Hospitals NHS Foundation Trust, Oxford, GBR; 6 Colorectal Surgery, AdventHealth, Tampa, USA; 7 Psychology, University of California, Riverside, USA

**Keywords:** acute generalised peritonitis, emergency abdominal surgery, endoscopic reduction, hollow viscous perforation, robotic colorectal surgery, sigmoid volvulus, transverse colon volvulus, volvulus, volvulus reduction, young female with volvulus

## Abstract

Transverse colon volvulus is a rare cause of potentially life-threatening large bowel obstruction accounting for less than 5% of colonic volvulus cases. Its diagnosis is often delayed due to nonspecific clinical features and its rarity. We report two uncommon cases of transverse colon volvulus. The first case involves a 20-year-old female who presented with acute abdominal pain and distension. The second case is a 67-year-old female with a one-week history of progressive constipation and abdominal discomfort. Computerized tomography aids in the diagnosis and definitive management requires surgical resection. Prompt diagnosis and treatment are critical due to the risk of ischemia and perforation.

## Introduction

Colon volvulus results in twisting of a bowel segment around its mesenteric axis, leading to obstruction and potential ischemia [1]. While sigmoid and cecal volvuli are relatively common, volvulus involving the transverse colon is exceedingly rare. It has been estimated to affect one in 85 patients according to Halabi et al. [2], 0.93% of all patients with colonic volvulus reported by Huerta et al. [3] and <5% of all colonic volvulus according to Lau et al. [4]. Volvulus results in mechanical obstruction and vascular compromise with early venous outflow obstruction resulting in bowel edema followed by arterial inflow compromise leading to mucosal ischemia and bacterial translocation and later transmural ischemia with perforation and peritonitis [5]. Female patients in the second or third decades of life are more likely to be affected [6]. The clinical presentation can vary significantly and is nonspecific, leading to missed or late diagnosis, increasing the risk for bowel infarction, necrosis, perforation, peritonitis and death and constitutes a surgical emergency [7].

## Case presentation

Case 1

**Figure 1:**
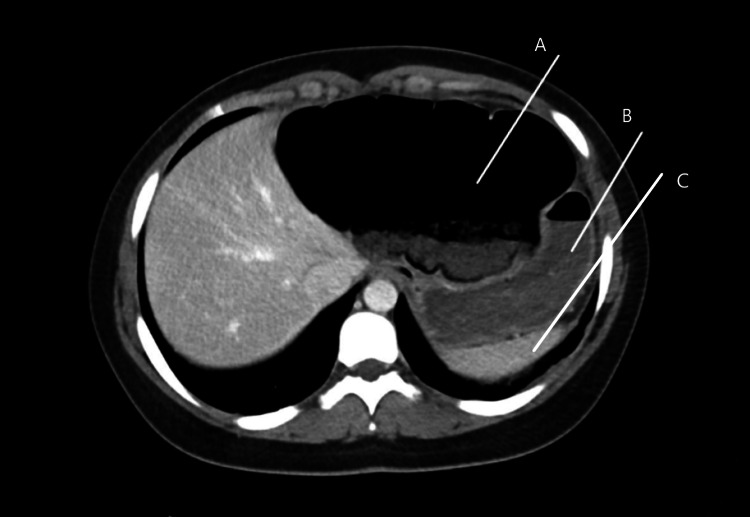
Computerized Axial Tomography, case 1. A-Distended transverse colon B-Decompressed and displaced stomach C-Spleen

**Figure 2:**
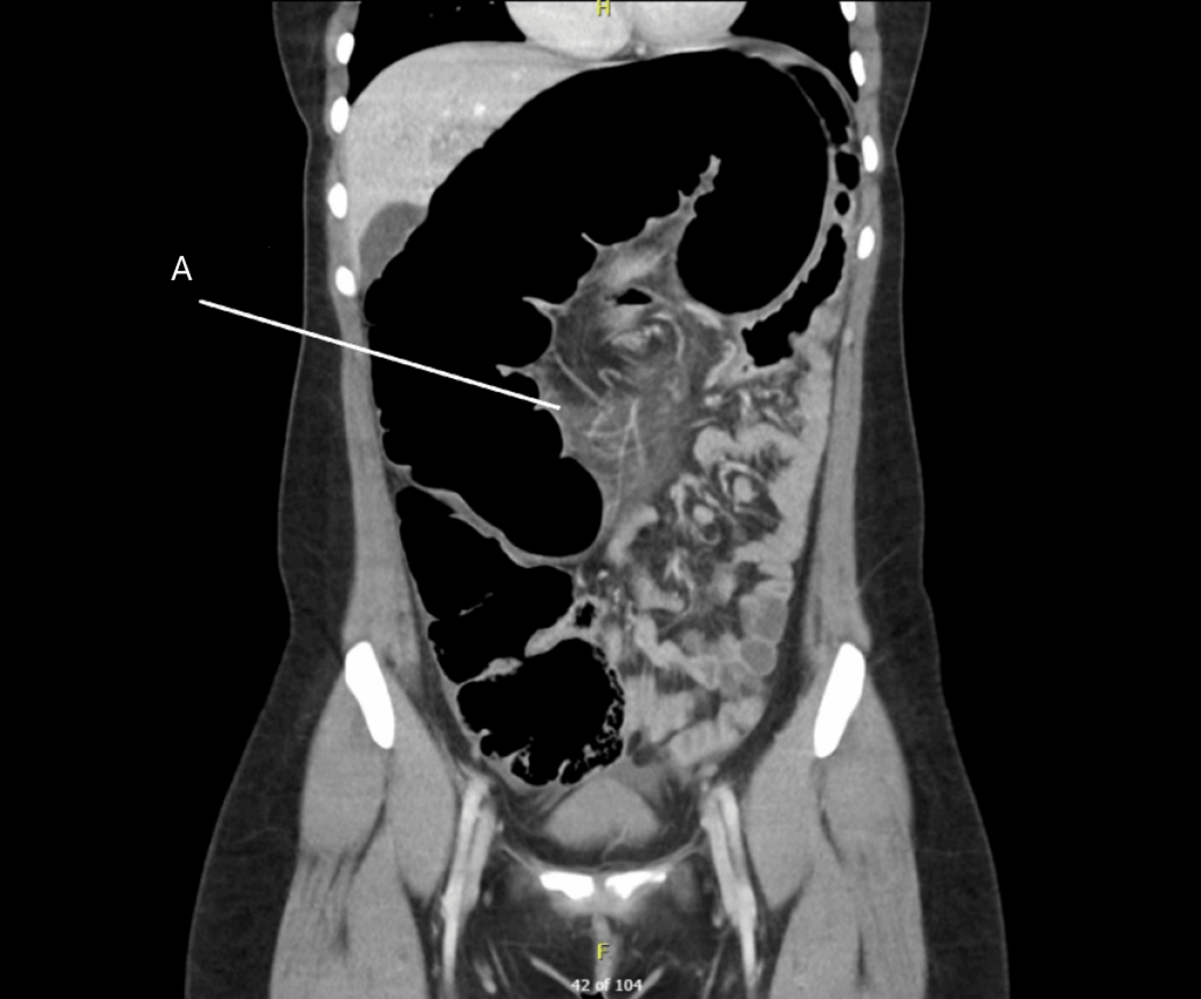
Computerized tomography (Coronal), case 1. A-Image raising concerns for swirl within the mesenteric root.

A 20-year-old Hispanic female presented to the emergency department with acute onset, severe midabdominal pain. She denied any prior surgical history, substance use, or smoking, although she did report a longstanding issue with chronic constipation. Approximately 15 minutes after consuming a burrito at lunch, she began experiencing escalating abdominal discomfort, which progressed over the following four hours to include marked distention, nausea, and emesis. On examination, the patient was hemodynamically stable, afebrile with abdominal distention, tenderness on deep palpation, absence of overt peritoneal signs, and auscultation revealed hyperactive bowel sounds. Laboratory findings revealed elevated WBC 13.47 (normal 4-11 X 10*3/uL), mild hyperglycemia with a glucose level 118 (normal 70-99 mg/dL), otherwise unremarkable. Abdominal computed tomography with intravenous contrast demonstrated a significantly dilated large intestine with a clear transition zone localized to the mid-abdomen. Additionally, concerns were raised regarding a potential swirling pattern of the mesenteric vessels as illustrated in Figures 1, 2.

**Figure 3:**
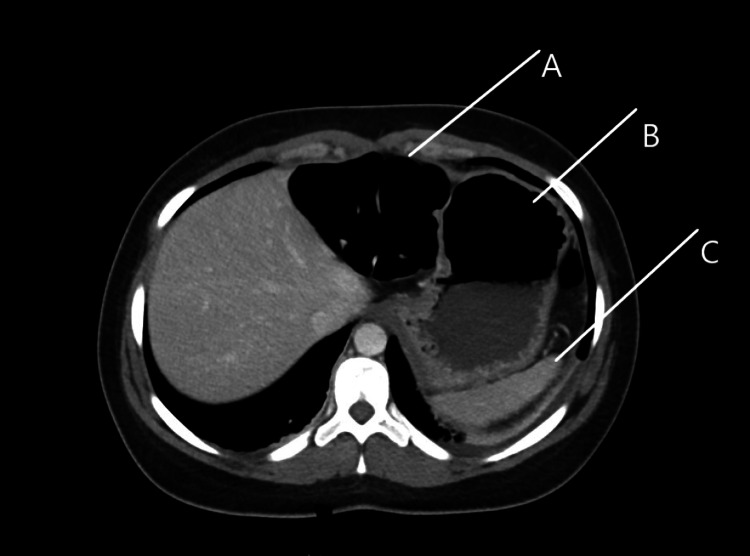
Computerized Axial Tomography upper abdomen, case 1. Image showing less distended transverse colon and relatively decompressed stomach: A-Decompressed transverse colon B-Relatively decompressed stomach C-Spleen

Initial management of provisional diagnosis for sigmoid colon volvulus raised with the CT findings involved colonoscopy decompression with rectal tube placement. The patient's symptoms improved without complete resolution, prompting reassessment on hospital day two and review of the CT with the radiologist and the diagnosis of transverse colon volvulus was raised. A follow-up contrast-enhanced abdominal CT scan confirmed substantial colonic decompression facilitated by the rectal tube, along with mild-to-moderate residual distention and diminishing evidence of mesenteric swirling (Figure 3).

Robot-assisted resection of the redundant transverse colon was performed as displayed in Video 1.

**Video 1:** Robot-Assisted Resection of The Transverse Colon, case 1. Resection of transverse colon due to volvulus using the Davinci Xi platform.

The patient did well and was discharged from the hospital on post-operative day three. No procedure-related complications were observed within the subsequent 90-day follow-up period.

Case 2

A 67-year-old African American woman presented emergently to a rural medical facility with a primary complaint of complete absence of bowel movements for one week. Her extensive past medical history was notable for idiopathic pulmonary fibrosis, systemic sclerosis (scleroderma), hypothyroidism, gastroesophageal reflux disease (GERD), irritable bowel syndrome (IBS), as well as previous cholecystectomy and hysterectomy many years prior to her presentation. Despite the prolonged constipation, she reported no accompanying gastrointestinal symptoms such as nausea, vomiting, or abdominal pain until the gradual onset of bloating over the course of the week. Her medication regimen included multiple inhaled corticosteroids: mometasone-formoterol, budesonide, and fluticasone. On physical examination, the patient was hemodynamically stable, afebrile, the abdomen was markedly distended and demonstrated diffuse tenderness on deep palpation in the absence of peritoneal signs. Laboratory findings revealed mild hyponatremia with a sodium of 131 (ref. 135-145 mmol/L), hypokalemia with a potassium of 3.1 (ref. 3.2-5.2 mmol/L), and an anion gap of 11 (ref. 6-8.3 g/dL). 

**Figure 4:**
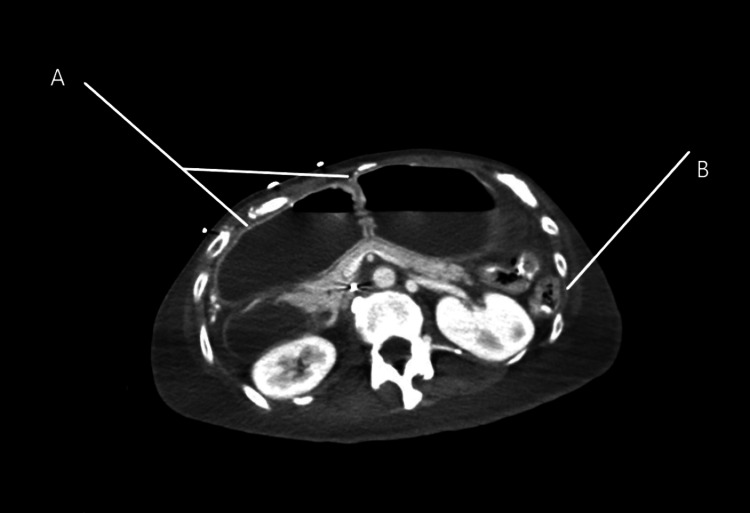
Abdominal CT, axial section, case 2. A-Dilated large bowel loops B-Decompressed descending colon

**Figure 5:**
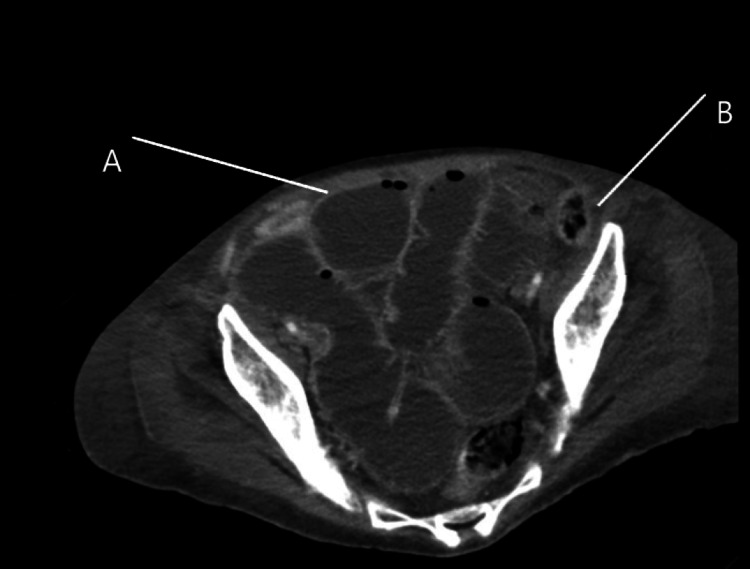
Computerized Axial Tomography Pelvis, case 2. A-Distended small bowel loops. B-Decompressed descending colon.

A contrast-enhanced CT scan of the abdomen revealed significant distention of the ascending and transverse colon with decompressed descending colon (Figure 4) and small bowel obstruction picture (Figure 5). 

Given radiographic suspicion, a colonoscopy decompression of a presumptive sigmoid volvulus was undertaken, followed by rectal tube placement. The patient was subsequently admitted for elective sigmoid resection during the same hospitalization. Due to persistent abdominal discomfort and consideration of her significant comorbid conditions, she was transferred from the rural facility to our tertiary care center. The patient underwent exploratory laparotomy via a midline incision. Intraoperative findings revealed extensive darkening of the cecum and right colon with imminent risk of perforation, in conjunction with severe distention of the twisted transverse colon. Notably, the proximal transverse colon exhibited dense adhesions at the prior cholecystectomy site, forming an axial fixation point around which volvulus had occurred. Surgical management included detorsion of the affected bowel loops followed by an extended right hemicolectomy with end ileostomy formation. The postoperative course was largely uneventful, aside from transient hypokalemia and hyponatremia, both of which were corrected with medical management. The patient achieved adequate recovery milestones and was discharged on postoperative day eight. No procedure-related complications were observed within the subsequent 90-day follow-up period.

## Discussion

Colonic volvuli constitute 5% of all cases of intestinal obstruction, 80% of which are related to sigmoid colon and 18% cecum, the reminder 2% involves the transverse colon [8]. Dilation of the colon as a result of chronic constipation is a possible cause of transverse colon volvulus with ensuing redundancy and non-fixation as a pathophysiology [9,10]. Previous abdominal surgery, history of volvulus, congenital malformations, chronic constipation, pseudomembranous colitis, and pregnancy are all risk factors that predispose to the onset of transverse colon volvulus [11]. It has been reported following colonoscopy and believed to be secondary to insufflation as a result of a non-fixed/mobile segment [12]. Differentiating transverse colon volvulus from cecal or sigmoid colon volvulus clinically and radiologically is challenging, and many are diagnosed intraoperatively. Computerized tomography findings that can aid in the diagnosis includes abrupt narrowing of the colon at the splenic flexure, crossing of the superior mesenteric vessels and whirl sign can be seen in all colonic volvuli and distended loop with tapering at both ends in sigmoid valvules, small bowel dilation and ileocecal twist in cecal volvulus, mesocolon twist that can mimic gastric or sigmoid volvulus in transverse colon volvulus [13]. Barium enema reveals a bird’s beak at the rectosigmoid in case of sigmoid volvulus, and a similar finding in the right colon in case of cecal volvulus and an abrupt cut off in the transverse colon in case of transverse colon volvulus [14]. Colon decompression in cecal and sigmoid volvulus is an initial treatment option, but it is not recommended in transverse colon volvulus due to the risk of failure and consequential delays in operative management and necrosis [15]. High risk of recurrence and death as a result of detorsion and colpopexy has been reported and should be avoided [16]. While the mortality rate for sigmoid colon volvulus is up to 21% and 10% for cecal volvulus, it is higher for transverse colon volvulus and is up to 35% [17,18]. Resection of the pathologic segment with stoma formation or primary anastomosis is the preferred option [11].

## Conclusions

Volvulus of the transverse colon is a rare cause of bowel obstruction that pauses a diagnostic challenge and is associated with high mortality risk. Early recognition with surgical resection preventing ischemia necrosis and perforation is key for successful outcome.
